# Psychocardiological assessment in the acute phase of the takotsubo syndrome: Somatic and depressive disorders, resilience and illness perception

**DOI:** 10.1007/s00508-021-01957-1

**Published:** 2021-10-20

**Authors:** Valerie Weihs, Edita Pogran, Evelyn Kunschitz, Wolfgang Weihs, Erika Prinz, Christiane Eichenberg, Jutta Fiegl, Oliver Friedrich, Kurt Huber

**Affiliations:** 1 Department of Orthopedics and Trauma Surgery, Medical University of Vienna, Vienna, Austria; 2 3rd Medical Department, Cardiology and Intensive Care Medicine, Clinic Ottakring, Vienna, Austria; 3 II. Medical Department for Cardiology, Takotsubo-Ambulanz, Hanusch Hospital, Vienna, Austria; 4 Department of Cardiology, Hospital Graz II, Graz, Austria; 5 Clinic of Internal Medicine II, Department of Cardiology, Paracelsus Medical University, Salzburg, Austria; 6 Institute for Psychosomatic, Medical Faculty, Sigmund Freud University, Vienna, Austria; 7 Faculty of Psychotherapy Science, Sigmund Freud University, Vienna, Austria; 8 Karl Landsteiner Institute for Scientific Research in Clinical Cardiology, II. Medical Department for Cardiology, Hanusch Hospital, Vienna, Austria; 9 Medical Faculty, Sigmund Freud University, Vienna, Austria

**Keywords:** Stress-induced cardiomyopathy, Stress profile, Coping strategies, Psychosomatic disorders

## Abstract

**Objective:**

To analyze the psychocardiological profile and the clinical characteristics in the acute phase of takotsubo syndrome (TTS).

**Methods:**

Prospective multicenter cohort study of TTS patients evaluating the clinical characteristics as well as the prevalence of somatic, depressive, panic, stress and anxiety disorders. Assessment of illness perception and resilience in the acute phase of the syndrome.

**Results:**

All 27 evaluated TTS patients were female with a mean age of 68 years (±11.4 years). The apical type of TTS was found in 60% of patients, followed by the combined type of TTS in 30% of patients. Main clinical symptom leading to hospital admission was chest pain in nearly 80% of patients. An ST-segment elevation mimicking acute myocardial infarction was found in 44% of patients and T wave inversion in 26% of patients. An endogenous (emotional) stress event was found in 17 patients (63.0%), an exogenous (physical) stress event in 5 patients (18.5%) and a combined stress event in 2 patients (7.4%). In 11.1% of patients (*n* = 3) no stress event could be found. Moderate to high levels of illness threatening were found in 48% of patients and low to moderate resilience scores were found in 40% of patients. Somatic disorders were found in half of the patients (56%) followed by depressive disorders in 26% of patients.

**Conclusion:**

Moderate to low resilience scores and moderate to high levels of illness threatening can be seen in the acute phase of TTS, reflecting the severity of the experience as an adverse life event. Patients suffering from TTS present in the acute phase with a high prevalence of somatic disorders and relatively high prevalence of depressive disorders.

## What is already known about this subject?

- Little is known about the underlying pathological mechanisms in patients with Takotsubo Syndrome (TTS). There are some recent studies demonstrating that there are specific alterations in neurological response and sympathetic activation after emotional stimuli in TTS patients suggesting the importance of the brain-heart interaction.

## What does this study add?

- Decreased resilience scores were found in about 40% of TTS patients in the acute phase of this syndrome.
- This might reflect a decreased ability to cope with this event and may lead to the reminiscence of the TTS as a threatening adverse life event, which can additionally be seen in moderate to high levels of illness threatening in these patients.
- Moderate to low resilience scores in TTS patients are on the one hand additional trigger factors for the development of the TTS and on the other hand potential prognostic factors for the recovery from this syndrome.

## How might this impact on clinical practice?

- As TTS seems to be accompanied by an activation of the sympathetic nervous system, decreased coping strategies in the acute phase of the syndrome might help to explain the pathomechanism of this syndrome. This may lead to improved therapeutic interventions, especially in the field of psychocardiology.
- Based on these findings, a multidisciplinary assessment of TTS patients with structured interviews by qualified psychologists addressing the involvement of emotional events in TTS patients with more accuracy has been initiated and is still ongoing.

## Background

TTS is a transient clinical syndrome mimicking acute myocardial infarction (MI) with comparable acute adverse events. It is named after the typical shape of the left ventricle in the acute phase of the syndrome assembling the pot used for trapping octopus in Japan—takotsubo. TTS is also named broken-heart-syndrome, transient apical ballooning syndrome, ampulla cardiomyopathy or stress-induced cardiomyopathy. The TTS typically affects older women and resolves within a few weeks. An emotional or physical trigger event might precede the syndrome [1–18].

As trigger factors physical triggers and emotional triggers are found in the same proportion, whereas in up to 30% of patients no trigger event can be found. Emotional (endogenous) trigger events can be acute unexpected emotional stress, accidents, death/funeral of loved ones, arguments or excessive alcohol consumption. Physical (exogenous) stress events can be exacerbation of an underlying comorbidity, an operative procedure or exceptional physical activity [6–10, 12, 13, 16, 19–22].

As the TTS often seems to be triggered by severe acute emotional or physical stress, detection of patients coping strategies, stress profile and underlying risk factors might give better insight to the underlying disorders of this disease.

The aim of this study was the analysis of the characteristics of TTS patients and their psychocardiological profile to gain more information about underlying risk factors, the stress profile, coping strategies and potential pathological mechanisms.

## Methods

### Design

The study was designed as a prospective, multicenter cohort study and took place between June 2013 and September 2018. After obtaining all institutional research ethics board approvals and signed informed consent a total of 27 consecutive patients with the proven diagnosis of TTS were enrolled prospectively in this study.

### Study sample

Overall, 37 patients admitted to the participating centers were prospectively enrolled between June 2013 and September 2018. All participating patients underwent coronary angiography. Of the patients two had to be excluded, one due to significant stenosis of coronary arteries in coronary angiography and one due to retrospective inclusion, and another 8 patients had to be excluded due to the lack of essential data leading to remaining 27 patients with a proven diagnosis of TTS.

### Clinical assessment

The main characteristics of patients were documented in each participating center according to a specific data form for acquisition of patients with TTS: including demographic and clinical parameters, 12-lead electrocardiograph (ECG) results on admission, evaluation of coronary arteries, determination of regional wall motion abnormalities (WMA) in the acute phase either with echocardiography or levocardiography, laboratory parameters and in-hospital complications and clinical outcome.

### Psychosomatic assessment

As TTS seems to be triggered by severe acute emotional or physical stress, detection of patients coping strategies, stress profile and underlying risk factors may give another insight into the underlying disorders of this disease.

A standardized questionnaire to gain more information about underlying risk factors, the stress profile, coping strategies and potential pathological mechanisms was administered within the acute phase of hospitalization due to the TTS. The study questionnaires included:

- Brief Illness Perception Questionnaire (B-IPQ) to detect emotional and cognitive representations of illness or health threat [23]. For further interpretation the score of the B‑IPQ was divided into three groups: low level of threatening illness perception (scores from 0–27), moderate level of threatening illness perception (scores from 28–55) and high level of illness perception (scores > 55).
- PHQ‑9 depression scale from the Patient Health Questionnaire (PHQ) for detection of depressive disorders [24] designed to diagnose and grade depression disorders. The questionnaire focuses on the nine diagnostic DSM-IV criteria for depression disorders. All items score between 0 (not at all) and 3 (every day). As a measure for severity of depression the score ranges between 0 and 27. It has been shown to have reasonable sensitivity and specificity for patients with coronary artery disease [25, 26].
- PHQ-15 somatic symptom scale from PHQ for the analysis of somatic disorders [27]
- Panic module, anxiety module and the stress module from PHQ‑D for detection of panic or anxiety disorders [28]
- Brief resilience scale (RS-13) to detect the patients’ ability to cope with the experienced stress [29].

### Inclusion criteria

Only patients older than 18 years who have signed the informed consent were included in this study.

Patients with following criteria adapted to the Mayo Clinic criteria for TTS were analyzed:

- Transient WMA of the left ventricular segments extending beyond a single epicardial vascular distribution.
- New electrocardiographic changes (ST/T changes) and/or typical symptoms indicative for myocardial ischemia and/or release of specific myocardial necrosis marker.
- Absence of obstructive coronary disease.

### Exclusion criteria

Patients who were suspected to have another disease other than TTS, such as acute myocardial infarction (AMI) with occluded or significantly narrowed epicardial coronary artery disease, hypertrophic cardiomyopathy, pheochromocytoma as well as recent significant brain trauma or intracranial bleeding were excluded from this study.

### Patient involvement

This research was done without patient involvement. Patients were not invited to comment on the study design and were not consulted to develop patient relevant outcomes or interpret the results. Patients were not invited to contribute to the writing or editing of this document for readability or accuracy.

### Statistical analysis

Continuous variables are presented as means and standard deviations or medians and interquartile ranges. Categorical variables are provided with percentages. Descriptive statistics were used for demographic variables and clinical characteristics. Types of TTS, patients’ characteristics and risk factors, patients’ stress profiles and stress events were examined. For detection of associations between qualitative variables a χ^2^-test was performed. For comparison between categorical and continuous variables the Student’s t‑test was done. A two-sided *P* value of less than 0.05 was considered to indicate statistical significance. All data manipulation and statistical analysis was performed using IBM® SPSS® statistics software for Macintosh, version 25.0 IBM Corp., Armonk, NY, USA.

## Results

### Clinical results and patient characteristics

The mean age was 68 years of age (from 42 to 85 years of age), all patients were female. Most patients had low educational status (51.9%) and were unmarried (25.9%). The main symptom leading to hospital admission was chest pain (*n* = 21; 77.8%) and/or dyspnea (*n* = 15; 55.6%) followed by nausea/vomiting (*n* = 4; 14.8%) or syncope in one patient. All patients showed slight to moderate elevation of the cardiac biomarkers. The initial diagnosis leading to coronary angiography was ST-segment elevation myocardial infarction (STEMI) in 10 patients (37.0%), non-ST elevation myocardial infarction (NSTEMI) in 9 patients (33.3%), TTS in 4 patients (14.8%) and unstable angina pectoris (AP) in 2 patients (7.4%). ST-segment elevations were seen in 12 patients (44.4%) followed by T wave inversions (*n* = 7; 25.9%), pathological Q waves (*n* = 3; 11.1%) and ST-segment depression (*n* = 2; 7.4%) on admission. Concomitant cardiovascular risk factors were seen in 85.2% of patients (*n* = 23): arterial hypertension (*n* = 17; 63.0%), hyperlipidemia (*n* = 17; 63.0%), type 2 diabetes mellitus (*n* = 7; 25.9%), active smoker (*n* = 4; 14.8%), history of smoking (*n* = 4; 14.8%) or positive family history of CAD (*n* = 4; 14.8%) (Table 1).

**Table 1:** Clinical results and patient characteristics

**Clinical assessment**	
**Age (years)**	68.0 ± 11.4 (42–85)
**Cardiovascular risk factor**	85.2%
**AHT**	63.0%
**Hyperlipidemia**	63.0%
**Diabetes mellitus II**	25.9%
**Active smoker**	14.8%
**History of smoking**	14.8%
**Family history of CAD**	14.8%
**Symptoms**	
*AP*	77.8%
*Dyspnea*	55.6%
*Nausea/vomiting*	14.8%
**ECG changes on admission**	
*ST-segment elevation*	44.4%
*T wave inversion*	25.9%
*Pathological Q waves*	11.1%
*ST-segment depression*	7.4%
**Cardiovascular complications**	33.3%
**Type of TTS**	
*Apical*	59.3%
*Combined*	29.6%
*Midventricular*	7.4%
*Basal*	3.7%
**Right ventricular involvement**	18.5%

*AHT* arterial hypertension, *CAD* coronary artery disease, *AP* angina pectoris, *ECG* electrocardiogram, *TTS* takotsubo syndrome

Cardiovascular complications were found in 33.3% of patients: 4 patients (14.8%) suffered from pulmonary edema, 2 patients (7.4%) from cardiac arrhythmias and 1 patient was in cardiogenic shock (Table 1). One patient died of the disease. Median length of hospital stay was 9.3 days (range from 2 to 29 days).

### Regional wall motion abnormalities and echocardiographic assessment

The apical type of TTS was documented in most patients (*n* = 16; 59.3%) followed by the combined type of TTS (*n* = 8; 29.6%), the midventricular type of TTS (*n* = 2; 7.4%) and the inverse type of TTS in one patient as assessed by acute echocardiography (*n* = 13) or levocardiography (*n* = 14). Hypokinesia of the affected left ventricular segments was seen in 6 patients (22.2%), akinesia in 20 patients (74.1%) and dyskinesia in 1 patient. Abnormal systolic function could be seen in 20 patients (74.1%): half of them showed a moderately (left ventricular ejection fraction (LVEF) from 35 to 39%) to severely reduced (LVEF < 35%) left ventricular function, moderately reduced in 9 patients (33.3%) and severely reduced in 5 patients (18.5%). Of the patients 6 (22.2%) showed a mildly reduced (LVEF from 40 to 54%) left ventricular function. A normal systolic function (LVEF from 55 to 70%) was documented in 6 patients (22.2%). Mean systolic PAP was 38 mm Hg (range from 28 to 54 mm Hg), 5 patients (18.5%) showed right ventricular involvement in the acute phase of the disease which could only be seen in patients with apical dysfunction of the left ventricle (apical or combined type of TTS). Mitral valve pathologies could be seen in 10 patients (37.0%), aortic valve pathologies in 5 patients (18.5%) and tricuspid valve pathologies in 5 patients (18.5%). Moreover, in one patient with an apical type of TTS left ventricular outflow tract obstruction could be seen with midventricular obstruction and a thrombus formation.

### Psychosomatic assessment

**Fig. 1:**
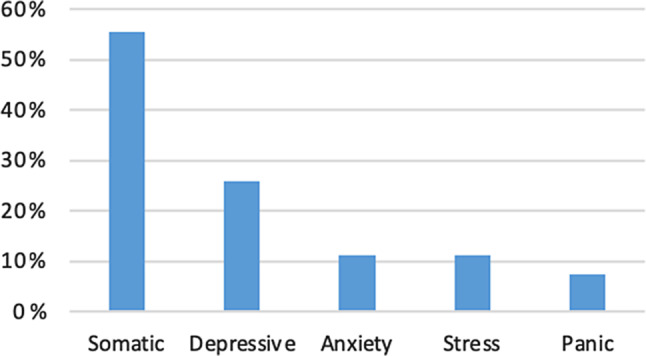
PHQ Assessment in TTS patients within the acute phase of the disease

An endogenous (emotional) stress event was found in 17 patients (63.0%), an exogenous (physical) stress event in 5 patients (18.5%) and a combined stress event in 2 patients (7.4%). Moreover, in three patients (11.1%) no trigger event could be found. The self-reported impact of emotional stress or physiological factors on the disease was reported as 58.8% (SD 31.1%; range 5–100%) (Table 2). Patients with an emotional stress event presented significantly more often with symptoms of chest pain compared to patients with physical or combined stress events (*p* = 0.007). The self-reported support from the patients’ surrounding was documented as 67.8% (SD 30.6%; range 5–100%). Somatic disorders were found in half of the patients (55.6%), whereas depressive disorders were found in 25.9% of patients. Panic disorders and anxiety symptoms showed a low prevalence in our patient cohort, 7.4% and 11.1%, respectively (Fig. 1). Furthermore, three patients were documented with mild stress symptoms. Somatic or depressive disorders did not significantly differ depending on the type of stress event, type of TTS, educational level or level of illness threat or age. TTS patients with depressive disorders showed a higher percentage of additional anxiety disorders, but not statistically significant (*p* = 0.075).

**Table 2:** Psychosomatic assessment in TTS patients within the acute phase of the disease

**Psychosomatic assessment**	
**B IPQ**	39.3 ± 10.8 (19–61)
**Consequences**	5.8 ± 2.7 (1–10)
**Timeline**	5.4 ± 3.5 (1–10)
**Personal control**	5.5 ± 2.4 (2–9)
**Treatment control**	6.4 ± 2.7 (1–9)
**Identity**	4.7 ± 2.6 (1–10)
**Concern**	5.6 ± 2.9 (1–10)
**Understanding**	6.3 ± 2.9 (2–10)
**Emotional response**	5.3 ± 3.1 (1–10)
**Support from surrounding**	67.8% ± 30.6 (5–10%)
**Impact of emotional stress**	58.8% ± 31 (5–100%)
**Level of illness threat**	
*Moderate to high*	48.1%
*Low*	7.4%
**Educational level**	
*Low*	51.9%
*High*	14.8%
**Resilience**	69.0 ± 16.6 (23–89)
**Resilience level**	
*High*	37.0%
*Low to moderate*	40.7%

The mean level of resilience was at a moderate level (69 points; range from 23 to 89 points). Low levels of resilience were documented in 29.6% of all patients, moderate levels in 11.1% of patients and high levels were documented in 37.0% of all patients (Table 2). No differences in resilience levels were found with respect to the type of TTS, the presence of somatic or depressive disorders or with respect to the educational levels or age.

The mean of total illness perception score was at a moderate level (mean 39.27; SD 10.82, range from 19 to 61). Moderate to high levels of illness threatening were found in 48.1% of patients (Table 2). Interestingly, the levels of illness threatening did not differ among patients with and without cardiovascular complications. In all 8 dimensions of illness perception moderate levels were obtained, whereas the levels in dimension of treatment control (mean 6.38; SD 2.65; range from 1 to 9) and coherence (mean 6.31; SD 2.89; range from 2 to 10) were documented highest. Perceptions of identity and time were documented lowest. No differences in the dimensions of illness perception were found with respect to the type of TTS, the presence of somatic disorders or the educational level. Patients with depressive disorder reported significantly higher perceptions of emotional representation (*p* = 0.023). Patients with high levels of resilience reported higher perceptions of identity compared to patients with low levels of resilience (3.9 vs. 7.0), but were not statistically significant.

## Discussion

Our study contributes several new insights into what is already known about psychosomatic disorders in TTS patients.

First, we found a high rate of somatic disorders in TTS patients and a relatively high rate of depressive disorders in accordance with previous studies [30]. TTS patients with depressive disorders tend to suffer more likely in addition of anxiety disorders in contrast to TTS patients without depressive disorders but not statistically significant. Compare et al. demonstrated that TTS patients with emotional trigger factors are characterized by lower scores in emotional intelligence, as well as higher scores on metacognitive beliefs and emotional processing deficits. The markers negative beliefs about thoughts, cognitive confidence and beliefs about the need to control thoughts were significantly higher reflecting their dysfunctional metacognitive stance [31]. Although Lazzeroni et al. suggested a possible relationship between anxiety disorders and emotional cardiac frailty in TTS patients [20], stress and anxiety disorders showed a low prevalence in our patient cohort.

Second, this is the first study examining the resilience in TTS patients in the acute phase of the syndrome. We were able to demonstrate low to moderate levels of resilience in about 40% of TTS patients. These results are contrary to a recent study on resilience in TTS patients. Olliges et al. found higher than normal scores of resilience in TTS patients [32]. Considering that their TTS patients were examined nearly 5 years after the TTS diagnosis, our results suggest that the resilience scores, evaluated in the acute phase, are decreased in some TTS patients. These decreased resilience scores are on the one hand additional trigger factors for the development of the TTS and on the other hand potential prognostic factors for the recovery from this syndrome. Low resilience scores in TTS patients and therefore a decreased ability to cope with this event, may lead to the reminiscence of the TTS as a threatening adverse life event. High incidences of stressful life events could be observed in TTS patients [33]. Smeijers et al. suggested that there might be a hyperreactivity of the sympathetic nervous system in response to mental stress but no emotional hyperreactivity in TTS patients [21]. As TTS seems to be accompanied by an activation of the sympathetic nervous system, decreased coping strategies in the acute phase of the syndrome might help to explain the pathomechanism of this syndrome.

Third, this is the first study to detect illness perception levels in TTS patients. TTS patients tend to have moderate to high levels of illness threatening independent on the type of TTS, the presence of somatic disorders or the educational level. TTS patients with depressive disorders reported significantly higher levels of perception of emotional representation, and moreover, patients with higher resilience reported higher perceptions of illness identity. Although the rate of cardiovascular complications was relatively high, the experience of cardiovascular complication did not have an influence on the level of illness threatening.

## Limitations and strengths

Potential limitations to our study include the small sample size but the psychosomatic aspects have been evaluated with high accuracy. Furthermore, patient selection may have been biased due to the inclusion of patients in the acute phase of the index event. This may have been an additional stress factor on the one hand but on the other hand better disclosed an underlying psychosomatic disorder. As we were not able to include male patients presenting with TTS, our data only apply for women with TTS, which however reflects the situation that TTS affects mainly older women. Specific strengths of this study are the multidisciplinary approach to TTS and the timing of the psychosomatic evaluation within in the acute phase of the TTS.

### Conclusion and further investigations

Patients with TTS present in the acute phase with a high prevalence of somatic disorders and relatively high prevalence of depressive disorders. Moderate to low resilience scores in TTS patients are on the one hand additional trigger factors for the development of the TTS and on the other hand potential prognostic factors for the recovery from this syndrome. Low resilience scores may result in a retrospection of the TTS as a threatening adverse life event, which can additionally be seen in moderate to high levels of illness threatening in these patients. As these data are based on questionnaires a multidisciplinary assessment of TTS patients with structured interviews by qualified psychologists addressing the involvement of emotional events in TTS patients with more accuracy has been initiated and is still ongoing.
